# Characteristics Associated With Being Asked About Violence Victimization in Health Care: A Swedish Random Population Study

**DOI:** 10.1177/0886260520977836

**Published:** 2020-12-06

**Authors:** Johanna Simmons, Katarina Swahnberg

**Affiliations:** 1 Linköping University, Linköping, Sweden; 2 Linnaeus University, Kalmar, Sweden

**Keywords:** domestic violence, disclosure of domestic violence, revictimization, sexual assault, violence exposure

## Abstract

Recommendations to routinely question patients about violence victimization have been around for many years; nonetheless, many patients suffering in the aftermath of violence go unnoticed in health care. The main aim of this study was to explore characteristics associated with being asked about experiences of violence in health care and thereby making visible victims that go unnoticed. In this study, we used cross-sectional survey data from 754 men (response rate 35%) and 749 women (response rate 38%) collected at random from the Swedish population, age 25–85. Questions were asked about experiences of emotional, physical, and sexual violence from both family, partner, and other perpetrators. Only 13.1% of those reporting some form of victimization reported ever being asked about experiences of violence in health care. Low subjective social status was associated with being asked questions (adj OR 2.23) but not with victimization, possibly indicating prejudice believes among providers concerning who can be a victim of violence. Other factors associated with increased odds of being asked questions were: being a woman (adj OR 2.09), young age (24–44 years, adj OR 6.90), having been treated for depression (adj OR 2.45) or depression and anxiety (adj OR 2.19) as well as reporting physical violence (adj OR 2.74) or polyvictimization (adj OR 2.85). The main finding of the study was that only few victims had been asked questions. For example, among those reporting ≥4 visits to a primary care physician during the past 12 months, 43% reported some form of victimization but only 6% had been asked questions. Our findings underline the importance of continuing to improve the health care response offered to victims of violence.

## Background

Victims of intimate partner violence have been found to be reluctant to spontaneously confide in health care providers about their victimization, but are often reported to appreciate being asked about violence (Feder et al., 2006; Hathaway et al., 2002). Therefore, universal screening for intimate partner violence has been advocated for many years. Specifically, the U.S. Preventive Service Task Force (USPSTF) recommends screening women of reproductive age for intimate partner violence and providing those who screen positive to ongoing support services (Force, 2018). The World Health Organization (WHO; Shetty & Howe, 2013) and the Swedish National Board of Health and Welfare (2014) make similar recommendations about screening for intimate partner violence in health care: Universal screening in all health care settings is not endorsed, rather providers are recommended to ask about intimate partner violence when assessing health conditions that may be caused or complicated by violence (Shetty & Howe, 2013). Examples given of such conditions are mainly psychiatric (e.g., depression, anxiety, post-traumatic stress disorder [PTSD], sleep disorders, substance abuse and suicidality or self-harm) or involve symptoms that remain unexplained despite medical investigation (e.g., chronic gastrointestinal symptom, chronic pain, headaches, reproductive symptoms or adverse reproductive outcomes). Also, other signs such as an intrusive partner in consultations or repeat consultations with no clear diagnosis are mentioned (Shetty & Howe, 2013). In addition, the WHO notes that routine enquire might be considered in antenatal care and psychiatric care, while the Swedish guidelines stipulate that routine enquire should be used in the same situations (Shetty & Howe, 2013; Swedish National Board of Health and Welfare, 2014).

To ask patients when there is an indication of violence is the most commonly used strategy in Swedish health care to identify victims of violence (Swedish National Board of Health and Welfare, 2018). The obvious problem with such an approach is that there are no single symptoms or signs that are pathognomonic for violence. An indication of whether the “target population,” that is, victims of violence and those at high risk of victimization, are asked about violence would be if characteristics associated with victimization are also associated with being asked about violence.

Despite there being different opinions concerning if universal screening should be applied or not, there is an agreement that many victims go undetected in the health care system. However, many health care professionals are hesitant to question patients about violence. Frequently reported barriers include: time constraints, lack of provider training about intimate partner violence, and lack of effective interventions for the victims (Alvarez et al., 2016; Waalen et al., 2000). Other factors may be specific characteristics of the health care provider: one Swedish study found that female health care workers were more inclined to screen for intimate partner violence than their male counterparts (Lawoko et al., 2011), and others have reported higher rates of screening in studies mainly including nurses and lower rates in studies mainly including physicians (Alvarez et al., 2016). One review found overall screening rates between 2 and 53%, with most studies in the lower half of that range (Alvarez et al., 2016). In Sweden, only 50% of caregivers (mixed sample of physicians, nurses, nursing assistants, midwives, and others) were found to have screened at even one occasion during the last three months (Lawoko et al., 2011). Many studies concerning screening are conducted from the caregiver perspective, while patient and violence characteristics are often framed in the context of disclosure or help-seeking. However, patient and violence characteristics may also affect the caregiver’s likelihood of screening. Studies suggest that caregivers’ prejudicial beliefs and victim-blaming attitudes may influence screening practices (Alvarez et al., 2016).

When discussing screening for violence victimization in health care, the focus has primarily been on intimate partner violence. However, over the last few decades, there is a growing understanding that experiences of violence are intertwined and repeated victimization by different perpetrators over a life course is common (Hamby & Grych, 2013; Hamby et al., 2016; Simmons et al., 2014, 2015; Teaster, 2017). Studies on polyvictimization, first introduced as a concept in research concerning children and youth, have found that the number of victimizations experienced are a more important risk factor both for poor health and renewed victimization than any single form of victimization alone (Finkelhor et al., 2007; Turner et al., 2010). Furthermore, recent findings have shown that victims of childhood violence are more likely than non-victims to report violent experiences as older adults (Kong & Easton, 2018; Wang & Dong, 2019).

To improve the health care system’s identification of, and in extension response to, victims of violence, we need a better understanding of factors influencing the identification of victims in health care. In this study, we will investigate characteristics of those who have been asked about violence in health care and thereby also making visible those that have never been asked. More specifically, we will

1. Report prevalence of violence victimization in a Swedish population sample and explore the proportion of victims and non-victims that have been asked questions about violence by a health care professional. Also, among those being asked about violence: investigate what kind of professional and in what health care setting they had been asked as well as if it was a predominantly positive or negative experience.
2. Explore what specific characteristics of victims and/or violence are associated with having been asked about abuse by a health care professional and compare this to characteristics associated with reporting victimization.
3. Explore characteristics of violence among male and female victims reporting being asked questions about violence.

## Method

### Sample and Procedure

This study was part of a larger study concerning violence, ill-health, and health care responses conducted in Sweden in 2012. The procedure and sample have been previously described along with a thorough investigation of non-response bias (Simmons & Swahnberg, 2019). In short, we used the population register to contact a random sample of 2,200 men and 2,000 women, aged 25–85 from the population of Östergötland in southeastern Sweden. After two reminders, a completed questionnaire was returned by 754 men (35%) and 749 women (38%). Respondents could choose to answer the questionnaire either in paper format or online.

### Measurements

Because the overall purpose of the study was multifold, the questionnaire used included several parts. For this study, the following measurements were used:

#### Violence victimization.

The NorVold Abuse Questionnaire (NorAQ), including questions about emotional (3 items), physical (2 items), and sexual (4 items) violence. NorAQ has been validated using an interview as the gold standard in both a male and a female Swedish sample (Swahnberg, 2011; Swahnberg & Wijma, 2003). Items are presented in Table 1. Previously, the exact wording of the questions as well as the study methodology for the data collection has been compared to other Swedish studies measuring the prevalence of violence, among them one using the WHO’s Violence against Women instrument and one using the revised Conflict Tactic Scale (Lovestad & Krantz, 2012; Nybergh et al., 2013). Generally, the wording of the questions in NorAQ covers more severe forms of violence than the other instruments and was found to produce lower prevalence rates (Simmons & Swahnberg, 2019). Compared to the original NorAQ, the answering alternatives were slightly modified for this study. In the original version, age at victimization was included in the response alternatives. In this study, each question about victimization was instead first answered with “yes” or “no.” Those with positive answers were then asked to specify if the perpetrator was a “family member (not partner),” a “partner” (current or previous), or “other.” For each question about physical and sexual violence and for each kind of perpetrator, the respondent was also asked to specify the frequency of victimization (1–2 times, 3–5 times, 6–9 times, 10 or more). For each question about emotional violence, the respondent was asked to specify the duration of victimization for each kind of perpetrator (<6 months, 6–12 months, 1–2 years, >2 years). For the sake of this study, polyvictimization was defined as reporting either more than one kind of perpetrator, more than one form of violence, or the same kind of violence at least 3–5 times/6–12months. To be able to distinguish polyvictimization, a variable was created with the categories: (a) No violence or single victimization and (b) Polyvictimization.

**Table 1.** Prevalence of Victimization Among All Respondents (Women *n* = 747; Men *n* = 749).

	Women		Men		*P*-value
	*N*	%	*N*	%	
Emotional violence					
Systematically repressed, degraded, humiliated	121	16.4	84	11.3	.0043
Limited contact with others, controlled	68	9.2	48	6.5	.0523
Living in fear because of threats	57	7.7	37	5.0	.0323
Any emotional violence	142	19.2	107	14.4	.0131
Physical violence					
Hit with fist, hard object, kicked, pushed violently, beaten, thrashed, or similar	127	17.4	188	25.8	.000
Life-threatened, by, e.g., trying to strangle you, showing a weapon/knife, or similar	41	5.6	81	11.2	.000
Any Physical violence	142	19.5	218	29.9	.000
Sexual violence					
Sexual humiliation	18	2.5	5	.7	.006
Touched body parts other than genitals	77	10.6	15	2.1	.000
Touched or forced to touch genitals, used your body to satisfy him/herself sexually	85	11.7	19	2.6	.000
Put or tried to put penis or object in vagina, mouth, or rectum	51	7.0	3	.4	.00
Any sexual violence	129	17.7	27	3.7	.000
Kind of perpetrator					
Family	72	9.6	47	6.3	.018
Partner	119	15.6	37	4.9	.000
Other	151	20.2	234	31.2	.000
Polyvictimization					
No violence	499	66.8	480	64.1	.27
Single victimization	55	7.4	105	14.0	.000
Polyvictimization	193	25.8	164	21.9	.0769
Two or three forms of violence	121	16.2	74	9.9	.0003
Two or three kinds of perpetrators	82	11.1	46	6.2	.000

#### Being asked about violence.

The following question was used to assess if the respondent had ever been asked questions about victimization in health care: Has a health care professional ever asked you if you have ever been subjected to violence? Answering alternatives: (a) No; (b) I don’t remember; (c) Yes, in primary care; (d) Yes, in the emergency room; (e) Yes, in psychiatric care; (f) Yes, in maternal care; (g) Yes, in other specialist clinic; (h) Yes, when I was admitted to a hospital; and (i) Yes, in another place, namely, ________. For those who answered affirmatively, this was followed by: Who asked the question or questions about violence? (a) Physician; (b) Registered nurse; (c) Auxiliary nurse; (d) Midwife; (e) Counselor, Psychologist or Therapist; and (f) Other. Multiple affirmatory answers were possible, both for the question concerning where and the question concerning by whom questions had been asked. Respondents were also asked to think about the first time in health care that they were asked questions about violence victimization and report if they thought the experience was: (a) Only negative; (b) Predominantly negative, but with positive elements; (c) Predominantly positive, but with negative elements; and (d) Only positive.

#### Socio-demographic characteristics.

Respondents were asked questions about their background characteristics, and responses were grouped as follows: (a) Marital status: (i) partner/married = steady relationship, living partner, married and (ii) Single; (c) Income each month before taxes: (i) low = SEK 0–19,900, (ii) Middle = SEK 20,000–39,900, (iii) High = more than 40,000 (1,000 SEK = approximately 100 U.S. dollars in February 2020); (d) Main occupation in the latest 12 months: (i) Employed = Employed full or part-time, self-employed, (ii) On leave, student = Parental leave, Other leave, Student, Trainee, (iii) Unemployed, sick = Unemployed, assisted job, on sick leave, other, and (iv) Retired. Respondents were asked about their highest completed education: (a) University education and (b) No university education. Finally, respondents were grouped according to their and their parents’ country of origin: (a) Sweden = both respondent and his/her mother and father were born in Sweden and (b) World = respondent and/or one or both parents were born outside of Sweden.

#### Subjective social status.

Studies have suggested that subjective social status may be more consistently and stronger correlated with both psychological functioning and health-related factors than objective measures such as income level and education indicators (Adler et al., 2000). We therefore included a measure of subjective social status, consisting of a ladder with 10 rungs (Adler et al., 2000). Respondents were asked to place an X on the rung that they thought best represented their stand in society, based on the following instruction:

Think of this ladder as representing where people stand in our society. At the top of the ladder are the people who are best off, those who have the most money, most education, and the best jobs. At the bottom are the people who are the worst off, those who have the least money, least education, and the worst jobs.

#### Living habits.

Smoking was assessed with one question and responses were coded as: (a) not smoking = never smoked or previously smoked but stopped and (b) smoking sometimes or daily. One question was used to assess physical activity: In a typical week, how much time do you devote to moderate or very strenuous physical activity? Response alternatives were coded as: (a) Little = no time or <1h, (b) Moderate = 1–3h or 4–5h, (c) Much = 5h or more. A glass of alcohol was defined as 45 cl of medium-strong beer, 33cl of strong beer, 15 cl of wine, 8cl of fortified wine, or 4cl of liquor. Two questions were used to assess drinking habits: (a) How often do you drink alcohol (Score: Never = 0, 1 day/month or less = 1, 2–4 days/month = 2, 2–3 days/week = 3, 4–5 days/week = 4, 6–7 days/week = 5) and (b) If you drink alcohol, how many glasses do you drink on a typical day? (Score: 1–2 = 1, 3–4 = 2, 5–6 = 3, 7–9 = 4, 10 or more = 5). The score for the two questions about drinking was then added and grouped together into: (a) No alcohol/low alcohol consumption = 1–2 points, (b) Moderate alcohol consumption = 3–4 points, and (c) High alcohol consumption = 5 or more points.

#### Health-related factors.

Participants were asked how many visits they had made to a primary care physician during the latest 12 months (concerning own disease or symptoms). They were also asked if they had ever been treated in health care for: (a) Depression, (b) Anxiety, or (c) PTSD. Because there was a strong comorbidity between anxiety and depression, those variables were merged into one with the possible answers: (a) No depression or anxiety, (b) Anxiety, (c) Depression, and (d) Depression and anxiety.

### Statistical Analysis

#### Aim one.

Prevalence of victimization was calculated for men and women separately, and we tested for differences in proportions between the sexes using chi-square test in MedCalc for Windows, version 15.0 (2019). Descriptive statistics were used to investigate the total proportion of respondents ever being asked questions about violence as well as the health care setting in which they had been asked questions, who had asked them, and their satisfaction with the response given.

#### Aim two

To understand if the “target population” had been asked questions about violence, a comparison of background characteristics associated with violence victimization and with reporting being asked about violence was conducted. First, Pearson’s chi-square test was used to investigate the binary associations between the respondent’s background characteristics and being subjected to violence as well as being asked questions about violence in health care. Both victims and non-victims were included in analysis but those answering that they did not remember if they had been asked about violence (*n* = 65, 4.5%) were excluded from the analyses about being asked.

Thereafter, two hierarchical logistic regression were performed, one with reporting ever being exposed to violence and one with ever being asked about violence (regardless of victimization status) as dependent variable. The models were built based on the principle of parsimony, that is, if a variable or block of variables did not contribute significantly to the fit of the model, it was excluded.

In the first hierarchical logistic regression model, reporting violence victimization was used as the dependent factor. Background characteristics, living habits, and health-related factors found to be significant in bivariate analysis were entered into the model in separate steps and those that significantly improved the model were retained. Education (*p* = .57), occupation (*p* = .40) and subjective social status (*p* = .07) were excluded from the final model because they did not contribute to the fit of the model. The model fit was good with only .6% (*n* = 7) of cases having studentized residuals over two and none over 2.5. Cook’s distance had a mean of 0.01 indicating that there was no specific case that had undue influence over the data.

In the second hierarchical regression model, ever being asked about violence in health care was put the dependent variable. Background characteristics, living habits, and health-related factors significantly associated with being asked in the bivariate analyses were entered into the model in separate steps. Variables found not to improve the model at significant levels were excluded at the next step, including civil state (*p* = .08), occupation (*p* = .41) and smoking (*p* = .07). Thereafter violence characteristics were entered, kind of perpetrator (i.e., family, partner, or other) was entered together in one block, followed by the form of violence (i.e., physical, sexual, or emotional) as one block. Both blocks were found to contribute significantly to the model (*p* < .01 for both). Finally, to investigate if polyvictimization could explain any variance beyond what was explained by all violence characteristics separately, polyvictimization (i.e., reporting no/single form of violence or polyvictimization) was entered and found to contribute significantly to the model (*p* < .01). However, looking at the parameter estimates in the model, after including both forms of violence, kind of perpetrator, and polyvictimization, some of the variables were no longer significantly associated with the outcome variable (i.e., partner perpetrator *p* = .40, other perpetrator *p* = .75, family perpetrator *p* = .82, sexual violence *p* = .07, and emotional violence *p* = .99). Based on the principle of parsimony, to reduce the number of missing cases and to reduce multicollinearity, these non-significant variables were excluded from the final model. The model fit of the final model was good with 2.9% (*n* = 36) of cases having studentized residuals over two and 1% (*n* = 13) over 2.5. Cook’s distance had a mean of 0.08 implying no undue influence over the model from potential outliers. Multicollinearity was a concern beforehand but found to not be a problem in the final model (Maximum VIF = 1.70 and lowest Tolerance = .59 for physical violence and polyvictimization).

#### Aim three.

Descriptive statistics were used to explore the type of violence (i.e., sexual, physical, emotional, multiple types) as well as the kind of perpetrator (i.e., family, partner, other, multiple perpetrators) reported by the male and female victims who had been asked questions about violence in health care. We did not test for differences in proportions between the sexes because the study did not have sufficient power to detect such differences due to the low proportion of victims reporting ever being asked questions in health care.

### Ethical Considerations

For some respondents, answering the NorAQ might be difficult and it may trigger memories and flashbacks of violence victimization. Therefore, contact information for an independent therapist was provided in the letter accompanying the questionnaire. The study was approved by the regional ethical review board in Linköping (Register No. 2012/194-31).

## Results

### Aim One

Prevalence rates for violence victimization are presented in Table 1. Female victims reported more sexual violence (women 17.7%, men 3.7%, *p* < .001) and intimate partner violence (women 15.9%, men 4.9%, *p* < .001) than male victims. Physical violence (women 19.5%, men 29.9% *p* < .001) and violence from other perpetrators (women 20.2%, men 31.2%, *p* < .001) were more commonly reported by men. Reporting two or more forms of violence (women 16.2%, men 9.9%, *p* < .001) as well as two or more perpetrators (women 11.1%, men 6.2%, *p* < .001) was more common among women than men.

Among all respondents, 89.6% (*n* = 1,283) reported that they had never been asked questions about violence by a health care provider, 4.5% (*n* = 65) did not remember, 4.8% (*n* = 69) had been asked at one health care facility, and 1.0% had been asked at multiple (*n* = 15). Among those reporting at least one form of victimization, 78.8% (*n* = 391) had never been asked questions about violence, 10.5% (*n* = 52) had been asked at one facility, 2.6% (*n* = 13) had been asked at multiple facilities, and 8.1% (*n* = 40) did not remember.

**Figure 1.**
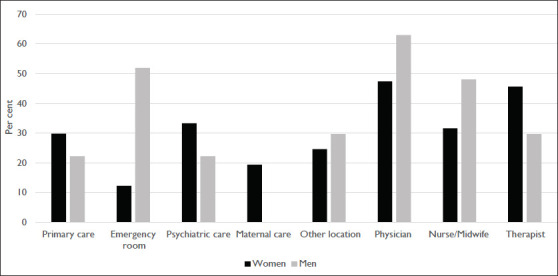
Health care facility where victims reported being asked about violence and the profession of those reporting to have asked the questions.

*Note*. Other location = In specialist care, when admitted to the hospital, other.

In total, significantly more women (*n* = 57, 8.2%) than men (*n* = 27, 4.0%) reported being asked about violence (*p* < .00; Table 2). Among those who had been asked questions, most men reported being asked in the emergency room (*n* = 14, 51.9%), while most women had been asked within psychiatric care (*n* = 19, 33.3%). Most often, it was a physician who had posed questions to both men (*n* = 17, 63.0%) and women (*n* = 27, 47.7%; Figure 1). The only statistically significant difference between the sexes concerning where questions had been asked and by whom was for more men than women being asked in the emergency room (men 51.9%, women 12.3%, *p* < .01; Figure 1.

**Table 2.** Background Characteristics of Respondents and Their Association With Reporting Victimization and Being Asked Questions About Violence.

			Any Victimization			Asked About Violence		
			Yes	No	*p*	Yes	No	*p*
	*n*	%	%	%	%	%	
Background characteristics								
Women	749	49.8	33.2	66.8	.27	**8.2**	**91.8**	**.00**
Men	754	50.2	35.9	64.1		**4.0**	**96.0**	
Age 25–44 years	424	29.1	**43.4**	**56.6**	**.00**	**11.9**	**88.1**	**.00**
Age 45–64 years	583	40.0	**35.9**	**64.1**		**5.9**	**94.1**	
Age >= 65 years	452	31.0	**24.8**	**75.2**		**1.7**	**98.3**	
Single	260	17.4	**48.4**	**51.6**	**.00**	**10.3**	**89.7**	**.01**
Partner/Married	1,232	82.6	**31.7**	**68.3**		**5.4**	**94.6**	
No university education	577	38.7	**32.2**	**67.8**	**.02**	5.5	94.5	.23
University education	914	61.3	**38.2**	**61.8**		7.1	92.9	
Employed	882	59.1	**36.7**	**63.3**	**.00**	**7.0**	**93.0**	**.00**
Paid leave	71	4.8	**40.8**	**59.2**		**9.4**	**90.6**	
Unemployed	121	8.1	**47.1**	**52.9**		**13.0**	**87.0**	
Retired	418	28.0	**25.1**	**74.9**		**1.9**	**98.1**	
Low income	589	40.3	34.1	65.9	.82	6.5	93.5	.34
Middle income	739	50.5	35.6	64.4		6.5	93.5	
High income	135	9.2	34.1	65.9		3.2	96.8	
COI Sweden	129	8.7	**33.6**	**66.4**	**.01**	5.9	94.1	.12
COI World	1,352	91.3	**44.4**	**55.6**		9.6	90.4	
Low subj soc status	535	37.6	**38.5**	**61.5**	**.03**	**9.4**	**90.6**	**.00**
High subj soc status	888	62.4	**32.7**	**67.3**		**4.5**	**95.5**	
Living habits								
Not smoking	1,240	85.2	**32.4**	**67.6**	**.00**	**5.4**	**94.6**	**.01**
Smoking	216	14.8	**50.0**	**50.0**		**10.3**	**89.7**	
No alcohol	169	11.6	**33.5**	**66.5**	**.00**	4.1	95.9	.28
Low alcohol	218	14.9	**31.7**	**68.3**		8.7	91.3	
Middle alcohol	769	52.6	**31.9**	**68.1**		5.6	94.4	
High alcohol	305	20.9	**43.3**	**56.7**		6.6	93.4	
Low physical activity	221	15.2	**41.1**	**58.9**	**.05**	5.5	94.5	.82
Medium physical activity	865	59.4	**32.8**	**67.2**		6.0	94.0	
High physical activity	371	25.5	**36.9**	**63.1**		6.8	93.2	
Health-related factors								
No visits PCP	482	34.9	**29.5**	**70.5**	**.00**	4.3	95.7	.08
1–3 visits PCP	625	45.3	**34.7**	**65.3**		7.7	92.3	
≥4 visits PCP	273	19.8	**42.8**	**57.2**		6.0	94.0	
No anxiety or depression	1,107	83.4	**30.1**	**69.9**	**.00**		**4.2**	**95.8**	**.00**
Anxiety	23	1.7	**47.8**	**52.2**		**17.4**	**82.6**	
Depression	108	8.1	**46.3**	**53.7**		**13.0**	**87.0**	
Anxiety and depression	90	6.8	**60.0**	**40.0**		**20.0**	**80.0**	
PTSD	32	2.2	**71.9**	**28.1**	**.00**	13.8	86.2	.09

*Note*. Missing cases: *n* = 0–127 (0–8.4%). Significant differences are written in bold.

COI = Country of origin: Sweden = Respondent and both parents born in Sweden; World = Respondent and/or one or two parents born outside of Sweden.

Subj soc = Subjective social, PCP = Primary care physician.

Nurse/midwife = Registered nurse, auxiliary nurse, midwife.

Therapist = Counselor, psychologist, therapist.

Most respondents who had been asked questions about violence considered it as being a positive (*n* = 44, 55%) or predominantly positive (*n* = 21, 26.3%) experience. Six respondents (7.5%) found it to be a predominantly negative experience, while nine respondents (11.3%) found it to be only a negative experience. No significant difference was found between men and women; therefore, we present aggregated data considering the small numbers in the categories reporting negative experiences.

### Aim Two

Binary analyses of respondents’ characteristics associated with exposure to violence and being asked questions about victimization are presented in Table 2. In the multivariate model the following factors were found to be significantly associated with reporting violence victimization: being young (age 25–44 adj OR 2.58; age 45–64 adj OR 1.82), being single (adj OR 1.73), country of origin World (adj OR 1.77), smoking (adj OR 1.62), high alcohol consumption (adj OR 2.22), medium physical activity (adj OR .72), ≥4 visits to primary care physician (adj OR 2.03) and ever reporting being diagnosed with depression (adj OR 1.88), depression and anxiety (adj OR 2.83) or PTSD (adj OR 3.33; Table 3). Background characteristics associated with ever having been asked questions about violence were: being young (age 25–44 adj OR 6.90; age 45–64 adj OR 3.19); being a woman (adj OR 2.09); reporting low subjective social status (adj OR 2.23); and ever been diagnosed with depression (adj OR 2.45) as well depression and anxiety (adj OR 2.16). The only violence characteristics associated with being asked questions were reporting physical violence (adj OR 2.74) and polyvictimization (adj OR 2.85; Table 4).

**Table 3.** Respondent Characteristics Associated With Reporting to Some Form of Violence Victimization.

	OR	95% CI	
Background characteristics			
Age 25–44 years	**2.58**	**1.84**	**3.62**
Age 45–64 years	**1.82**	**1.32**	**2.50**
Age >= 65 years	1		
Single	**1.73**	**1.24**	**2.42**
Partner/married	1		
Country of origin Sweden	1		
Country of origin outside of Sweden	**1.77**	**1.12**	**2.79**
Living habits			
Not smoking	1		
Smoking	**1.62**	**1.14**	**2.29**
No alcohol	1		
Low alcohol	1.14	.67	1.93
Middle alcohol	1.39	.88	2.19
High alcohol	**2.22**	**1.35**	**3.64**
Low physical activity	.89	.59	1.33
Medium physical activity	**.72**	**.54**	**.96**
High physical activity	1		
Health-related factor			
No visits primary care physician	1		
1–3 visits primary care physician	1.27	.96	1.69
≥4 visits primary care physician	**2.03**	**1.42**	**2.92**
No depression or anxiety	1		
Anxiety	1.77	.73	4.25
Depression	**1.88**	**1.22**	**2.90**
Depression and anxiety	**2.83**	**1.78**	**4.51**
No PTSD	1		
PTSD	**3.33**	**1.35**	**8.20**

*Note*. Model summary: –2 log likelihood 1498.2, Cox & Snell *R*-square = .11, Nagelkerke *R*-square .15. Significant differences are written in bold.

Included in analyses 1,268. Missing cases 235 = 15.6%.

**Table 4.** Respondent Characteristics and Violence Characteristics Associated With Being Asked About Violence Victimization in Health Care.

	OR	95% CI	
Background characteristics			
Women	**2.09**	**1.21**	**3.61**
Men	**1**		
Age 25–44 years	**6.90**	**2.78**	**17.11**
Age 45–64 years	**3.19**	**1.27**	**7.99**
Age > = 65 years	1		
Low subjective social status	**2.23**	**1.35**	**3.69**
High subjective social status	1		
Health-related factor			
No depression or anxiety	1		
Anxiety	2.47	.72	8.53
Depression	**2.45**	**1.20**	**5.04**
Depression and anxiety	**2.16**	**1.09**	**4.31**
Violence characteristics			
No physical violence	1		
Physical violence	**2.74**	**1.46**	**5.13**
No & Single victimization	1		
Polyvictimization	**2.85**	**1.53**	**5.30**

*Note*. Model summary: –2 log likelihood 463.158, Cox & Snell *R*-square = .10, Nagelkerke *R*-square .27. Significant differences are written in bold.

Included in analyses 1,367, Missing cases 158 = 11.6%.

Those not remembering if they had been asked about violence or not (*n* = 65) were excluded from the analyses.

### Aim Three

More male than female victims who had been asked questions about violence in health care reported “other” as the only perpetrator (women *n* = 14, 34.1%; men *n* = 15, 62.5%). Male victims who had been asked questions were most likely to report physical victimization only (*n* = 12, 50%) closely followed by two or more forms of violence (*n* = 11, 45.8%). Female victims were most likely to report two or more forms of victimization (women *n* = 21, 51.2%; Table 5).

**Table 5.** Violence Characteristics Reported by Male (*n* = 24) and Female (*n* = 41) Victims Who Had Been Asked Questions About Violence in Health Care.

		Women		Men	
		*n*	%		
Form of violence	Only emotional	8	19.5	1	4.2
	Only physical	7	17.1	12	50.0
	Only sexual	5	12.2	0	0
	2 or 3 forms	21	51.2	11	45.8
Kind of perpetrator	Only family	3	7.3	1	4.2
	Only partner	11	26.8	2	8.3
	Only other	14	34.1	15	62.5
	2 or 3 kinds	13	31.7	6	25.0

## Discussion

Not even 6% of the total sample and only 13% of those reporting victimizations remembered ever being asked questions about violence in health care. On the positive side, most respondents being asked were content with the experience.

### Sex

The kind of violence women and men are exposed to differ. As can be seen in Table 1, men report more physical violence and women more sexual violence. Also, women report more violence by intimate partners and men report more violence by other perpetrators. Being female was associated with being asked questions in health care. Considering that screening is generally recommended for intimate partner violence against women, it is not surprising that more women had been asked questions. Indeed, more than every fourth female victim who had been asked questions reported experiences of intimate partner violence only. However, not even 1 in 10 female respondents remembered ever being asked, while 1 in 3 had been victimized.

Among victims who had been asked questions, men reported physical violence and “other” as the perpetrator more often than women, further underling the different forms of violence men and women are exposed to. When medical attention is needed in the aftermath of physical violence, it is likely given in the emergency room due to physical injuries. In accordance with this, men were most often asked about violence in the emergency room. Women, on the other hand, reported being asked more often in psychiatric care and often by a therapist. This may indicate that the kind of violence women are exposed to is more often chronic in nature and may cause more long-term psychological ill-health. Another possibility is that health care professionals do not consider violence victimization as a reason for psychological ill-health among men as often as they do for women. However, it should be underlined that some men are victims of severe and chronic forms of violence, including intimate partner violence (Hines & Douglas, 2010a, 2010b). Previous studies have found that men are reluctant to talk about violence victimization in health care, in part due to experiences with or expectations of health care professionals portraying men only as the perpetrator and not as victims of violence (Douglas & Hines, 2011; Simmons et al., 2016). Altogether, more than every third man (35.9%) reported some form of victimization, but only 4.0% of the male respondents had been asked questions.

### Age

Younger age was associated with both reporting victimization and being asked questions about violence (Tables 3 and 4). Only 1.7% of older adults, age 65–85, had been asked questions about violence, though 24.8% reported victimization. Older adults are often overlooked as potential victims of violence. For example, interventions in health care for victims of intimate partner violence are increasingly common, but interventions for older adults are lagging. Moreover, there is a lack of evidence-based intervention strategies for this group (Dong, 2015; Du Mont et al., 2015; Ploeg et al., 2009). Consistent with this, the USPSTF found insufficient evidence concerning screening older adults for violence, because not enough studies have been conducted (Feltner et al., 2018; US Preventive Services Task Force, 2018). In Sweden, the government has encouraged municipalities to have a higher ambition and quality in their efforts to prevent, identify, and handle elder abuse (Ministry of Health and Social Affairs, 2014). Previously, older adults have been found to be less inclined to seek help after intimate partner violence than younger adults (Cho et al., 2017), making health care professionals’ ability to identify victims even more crucial. Improvements are necessary as shown by one study from the United States reporting that two-thirds of older adults suffering from severe physical violence were not reported to Adult Protective Services (Friedman et al., 2014). The study found that older adults not fitting into preconceived ideas concerning victims (i.e., younger elderly, males, blacks, and persons with alcohol/drug issues) were less likely to be reported to APS (Friedman et al., 2014).

### Social Status

Reporting low subjective social status was not associated with reporting victimization but with being asked about violence in health care. This study has a patient perspective and it is therefore not known if our measure of subjective social status correlates with health care provider’s perception of the respondent’s social status or not. One previous study found a weak correlation between a high level of perceived discrimination and lower subjective social status (Subramanyam et al., 2012). If the same is true for our sample, it might indicate that health care professionals hold prejudicial beliefs about who is subjected to violence and that this affects their decision to ask questions. In one review concerning screening for intimate partner violence, most providers (samples included a mix of profession) were found to have positive attitudes toward screening, but in a few cases negative attitudes were reported, for example, providers reporting that intimate partner violence was a problem only for a certain group (Alvarez et al., 2016).

### Health Factors and Living Habits

Risky health behaviors, that is, smoking and high alcohol consumption were associated with victimization but not with being asked questions. This confirms previous findings that victimization increases the odds of risky health behaviors such as smoking and binge drinking (Pengpid & Peltzer, 2020; Shetty & Howe, 2013). This might be due to poor coping and general ill-health in the aftermath of victimization. Also, high alcohol consumption may be a risk factor for violence. The association between physical activity and victimization was not as clear. Moderate physical activity was associated with lower odds of reporting violence. It might be that victims of violence either resign to physical inactivity or use excessive physical activity as a coping mechanism. A patient-provider discussion about health behaviors is encouraged in many health care settings and information about smoking, alcohol consumption, and physical activity is therefore often known to providers. When risky health behaviors are identified this should be considered an indication for asking about victimization.

Victimization was associated with ill-health, in the sense that victims reported more visits to primary care and had been diagnosed with mental ill-health more often than non-victims. Reporting depression or depression and anxiety were also associated with being asked questions about violence. Reporting only anxiety was associated with higher odds both for reporting violence and being asked questions, but this result was not significant. The lack of significance might be attributed to few respondents (*n* = 30, Table 2) reporting only anxiety. Similarly, Cheng reported poor mental health to be associated with disclosure of intimate partner violence to health care providers (Cheng & Lo, 2019).

Reporting PTSD or frequent visits to primary care was not associated with being asked questions. Lack of correlation for PTSD was unexpected as some form of trauma is a prerequisite for the diagnosis of PTSD. It might be that PTSD was mainly diagnosed in association with other forms of trauma such as accidents or natural disasters. Another possibility is that NorAQ does not adequately capture violence victimization outside of close interpersonal relationships, for example, acts of war, which may have caused PTSD.

Repeat visits to a primary care physician were correlated with victimization, indicating violence as a hidden reason for visits in health care. Among those reporting four or more visits to a primary care physician 43% reported some form of victimization but only 6% had ever been asked questions about violence. Likewise, though reporting mental ill-health was correlated with being asked about victimization, only a minority had been asked questions about violence. For example, 63% of those reporting being treated for anxiety and depression also reported exposure to violence, but only 20% had been asked questions about victimization. For depression only, the corresponding numbers were 46% for ever exposed and 13% for ever been asked.

### Kind of Violence and Type of Health Care Provider

Physical violence was the only single kind of violence that was positively correlated with ever been asked questions about violence in health care. Repeat physical violence has previously been associated with screening for intimate partner violence as well as for help-seeking among victims (Cheng & Lo, 2019; Cho et al., 2017). Considering that physical violence may lead to physical injury requiring medical care that cannot be overlooked, it is not surprising that it is more often paid attention to by health care professionals. However, emotional violence and sexual violence are well known to be correlated with ill-health and hence, need to be paid attention to in health care.

Reporting polyvictimization was found to be associated with being asked questions about victimization in health care. Considering the strong association between polyvictimization and both ill-health and repeat victimization in other studies, this is positive. It indicates that victims of the most severe forms of violence are more likely to be identified. As knowledge of the importance of polyvictimization for the health of victims increases, the health care response must follow. Many studies and health care interventions concern a specific form of violence, most often intimate partner violence. Although this makes sense in many ways, we need to start shifting the focus toward including also other forms of violence. Routine inquires with a broader perspective may enhance our response to victims, both by giving proper support and preventing future victimization.

Physicians were most commonly reported as the type of provider who had asked questions about violence. One previous Swedish study found that physicians and nurses were equally likely to screen for intimate partner violence (Lawoko et al., 2011), while an international review found higher screening rates in samples consisting of mainly nurses than in samples including mainly physicians (Alvarez et al., 2016). Our results should be interpreted with caution since they only represent descriptive data, we do not have knowledge about other factors that are known to affect screening practices, for example, education about violence (Alvarez et al., 2016).

### Limitations

This was a cross-sectional study of lifetime experiences of victimization and being asked questions about violence in health care. Although we expect that most participants have had some contact with the health care system during their life, we do not know when or how often or the temporal sequence between victimization and visits to health care. Taking the lifetime perspective is relevant as victimization for many is a chronic condition. However, it also increases the risk of recall bias. Less severe forms of victimization might not have been reported, and respondents might have forgotten being asked questions about violence in health care. However, participants were given the opportunity to answer “do not remember” concerning being asked questions in health care, an opportunity taken by few. The study did not have sufficient power to test for sex differences concerning violence characteristics among victims reporting being asked questions. However, numbers reported in Table 5 strongly indicate sex differences concerning violence characteristics in this group, which should be explored further.

Our questions concerning being asked about violence in health care did not pertain to any specific form of violence. Hence, respondents who reported being asked about violence may have been asked about, for example, physical violence by a peer while living with experiences of childhood abuse or sexual abuse. Also, not being asked questions about violence does not necessarily equal not talking to a health care professional about victimization; victims might have raised the issue themselves.

Although most participants reported that being asked questions was a positive experience, we do not know what made it a negative experience for some. Furthermore, we do not know anything about the response or referrals given.

We interpret a report of low subjective social status as an indication that health care providers perceived the victim’s social status as low. However, we do not have any data concerning the provider’s perception and hence do not know how well the two perspectives correlate.

### Clinical Implications

According to a review by Alvarez et al. (2016), including samples with mixed professions, screening practices for intimate partner violence may be affected by beliefs by professionals that they can tell who was subjected to violence or at risk thereof. The factors associated with being asked questions in our multivariate analysis (i.e., younger age, female sex, and reporting depression or depression and anxiety) were also associated with exposure to violence, indicating that the target population was asked questions. However, low subjective social status was only associated with being asked questions, not with violence exposure. This finding indicates that health care professionals prejudice believes about who is a victim of violence influence who they decide to ask questions. When the WHO guidelines were formulated some members of the guideline development group raised concerns about only recommending screening when there were signs or symptoms of violence. One reason for this was that health care providers may not be familiar with signs and symptoms of violence and therefore may stereotype patients and only ask those they believe can be victims of violence (Shetty & Howe, 2013). Our results support this apprehension and if true, this means that some victims will never be identified because they do not fit victim stereotype.

However, our main finding is the very low rate of respondents who had been asked questions in health care. Only 9% of the total sample had been asked questions about violence and among those reporting some form of victimization the proportion was 13%. Respondents with a history of depression and anxiety were more likely to have been asked about violence but even among them, being asked questions was uncommon. This indicates that the strategy taken by many Swedish health care institutions, namely, to ask when there is an indication of victimization, may not work. These are arguments for screening all patients for a history of violence. However, such recommendations have been in place for many years in the United States now. Nonetheless, research suggests that the achieved rate of screening is generally low, often around 10–20% (Alvarez et al., 2016; Sutherland et al., 2017). Likewise, a Cochrane review evaluated screening for intimate partner violence in health care settings and found that it increased the rate of identification, but the number was still low compared to estimates of prevalence of such violence (O’Doherty et al., 2014). This shows that policies concerning screening are not enough to identify victims. It is also not a prerequisite for identifying more victims; previous training and support for staff have been found to lead to higher rates of identification without universal screening (Feder et al., 2011).

In this study, most respondents reported that being asked about violence was a positive experience. Likewise, no harm could be identified from screening for intimate partner violence in the short run in either the Cochrane review or by the USPSTF, albeit insufficient evidence was found (Feltner et al., 2018; O’Doherty et al., 2014). However, the USPSTF did find that studies that only included brief interventions and/or provided information concerning where to refer patients were generally ineffective, for example, they did not show a reduction in prevalence of intimate partner violence or improvement in quality of life (Feltner et al., 2018). When asking about violence, it is vital that the health care system is ready to respond. However, in one Australian study, 40% of female victims of intimate partner violence did not think that they had received the help they needed in the health care system (Fanslow & Robinson, 2010), and female victims of sexual violence have indicated that help services may be helpful, but also harmful (Ullman, 2007). In a Swedish study of female victims of intimate partner violence, victims reported experiencing re-traumatization, uncaring behavior, and suffering in meeting with health care professionals (Pratt-Eriksson et al., 2014). A non-judgmental, nondirective, and individually tailored meeting with health care professionals who understand the complexity of intimate partner violence is what women desire (Feder et al., 2006).

Our findings support the idea of universal screening for violence in health care so that providers prejudicial believes does not affect who is asked questions. However, likely even more important is an increased general knowledge among health care providers about violence victimization, its health consequences, and how to respond. The health care system is an important entry point for assistance, but many other support services are likely to play critical roles for victims. In the Swedish national guidelines, it is very clear that when violence is detected, health care providers should cooperate with social services and non-governmental organizations to provide help to victims (Swedish National Board of Health and Welfare, 2014). Yet, in many cases, health care providers are not aware of the services offered by others and there are only limited examples in Sweden of programs where health care professionals and social services offer assistance in close cooperation. Perhaps the most effective way of responding to victims of violence would be to create outpatient clinics in collaboration between health care, social services, and non-governmental organizations. In addition to improving the response given to victims, this would give health care providers clear directives about where to refer patients, which would likely help to improve detection rates.

## Conclusion

Although interpersonal violence is a strong risk factor for ill-health, only few victims reported being asked about it in health care. Even victims reporting high health care use and health conditions well known to correlate with victimization, such as anxiety and depression, had only rarely been asked questions about violence. Provider’s prejudice believes about who can be a victim of violence may influence who is asked questions. One positive finding was that polyvictims, who may be most at risk for suffering in the aftermath of victimization, were more likely to report being asked about violence in health care. Taken together, there is a great need for improvements in the health care system’s identification and response to victims of violence. Improvements need to include system level, clinical level, and provider level factors as well as closer cooperation with social services and other support services outside the health care services.
